# Zolbetuximab for Unresectable and Metastatic Gastric and Gastroesophageal Junction Adenocarcinoma: A Review of Literature

**DOI:** 10.7759/cureus.75206

**Published:** 2024-12-06

**Authors:** Ananya Samanta, Arindam Ghosh, Monalisa Sarma

**Affiliations:** 1 School of Medical Science and Technology, Indian Institute of Technology, Kharagpur, IND; 2 Biochemistry, Dr. B.C. Roy Multispeciality Medical Research Centre, Indian Institute of Technology, Kharagpur, IND; 3 Subir Chowdhury School of Quality and Reliability, Indian Institute of Technology, Kharagpur, IND

**Keywords:** chemotherapy, cldn18.2-positive, gastric cancer, gastroesophageal junction adenocarcinoma, her2-negative, immunohistochemistry, monoclonal antibody, zolbetuximab-clzb

## Abstract

This article comprehensively reviews the working, efficacy, and safety profile of zolbetuximab. Zolbetuximab is a pioneering chimeric monoclonal antibody designed to target Claudin 18.2 (CLDN18.2), a tight junction protein frequently overexpressed in various gastrointestinal cancers, including gastric (G) and gastroesophageal junction (GEJ) adenocarcinomas. This drug has captured attention in the treatment of unresectable and metastatic G/GEJ cancer, especially in HER2-negative patients whose tumours express CLDN18.2. Zolbetuximab is a drug that binds to CLDN18.2, and its binding initiates an immune response that attacks and kills the cancer cells. It is typically co-prescribed with fluoropyrimidine and platinum-containing chemotherapy. The drug (formerly IMAB362), brand name Vyloy, was developed by Astellas Pharma, Tokyo, Japan. After multiple rounds of clinical trials, it was approved by the U.S. Food and Drug Administration (FDA) as a first-line treatment for locally advanced, unresectable cancer, establishing itself as a promising option for advanced G/GEJ cancers.

Studies reveal that it targets CLDN18.2 for the selective killing of cancer cells while sparing most healthy tissues. Zolbetuximab has demonstrated a significant improvement in progression-free survival (PFS) when combined with chemotherapy (like mFOLFOX6 or CAPOX). Also, it showed prolonged PFS compared to chemotherapy alone in previously untreated, CLDN18.2-positive, HER2-negative patients. Zolbetuximab has also shown improvements in overall survival (OS), regardless of whether the cancer progresses. This is a crucial benefit for patients with advanced G/GEJ adenocarcinomas. Additionally, zolbetuximab’s dual action triggers antibody-dependent cellular cytotoxicity (ADCC) and complement-dependent cytotoxicity (CDC). This enhances the immune system's ability to destroy cancerous cells and results in highly potent tumour destruction compared with chemotherapy alone.

Zolbetuximab is a relatively safe drug with a few adverse effects. The most frequently reported side effects were gastrointestinal, namely nausea, fatigue, vomiting, decreased appetite, diarrhea, neutropenia, anemia, and thrombocytopenia, potentially due to specific CLDN18.2 expression in the gastric mucosa. Its side effects are generally manageable, with no unexpected toxicity beyond those typically seen in patients receiving chemotherapy.

## Introduction and background

Gastric and gastroesophageal junction adenocarcinomas (G/GEJ) are malignancies originating from the epithelial cells lining the stomach or the junction where the esophagus meets the stomach. They represent significant global health challenges due to their high prevalence and associated mortality. Globally, gastric cancer ranks as the fifth most common cancer, with more than one million new cases diagnosed each year and 770,000 deaths recorded in 2023 [1]. It is most prevalent in East Asia (particularly in countries like Japan, South Korea, and China), where dietary and environmental risk factors contribute significantly to its high incidence [2]. Other regions with high gastric cancer rates include Eastern Europe and Central and South America. GEJ adenocarcinoma, though less common than gastric cancer, has seen rising incidence, particularly in Western countries. This increase is associated with factors like obesity and gastroesophageal reflux disease (GERD) [1].

Unresectable gastric/gastroesophageal junction (G/GEJ) adenocarcinoma is associated with Claudins (CLDNs), a family of key membrane proteins that play a crucial role in regulating tight junctions between epithelial cells. In normal tissues, CLDNs create barriers in the intercellular spaces of epithelial layers, controlling tissue permeability, paracellular transport, and signal transduction. Claudin18.2 (CLDN18.2), part of the CLDN family, is typically found in gastric mucosal cells in normal tissue, and its expression is frequently preserved in gastric cancer cells. CLDN18.2 is overexpressed in approximately 30-50% of gastric and GEJ adenocarcinomas. CLDN18.2-positive refers to the expression of the Claudin 18.2 protein on the surface of the cancer cell, making it a viable therapeutic target. CLDN18.2-positive status opens the possibility of using zolbetuximab as an emerging therapy that specifically targets this protein, which is present in a significant proportion of G/GEJ cancers [3].

The identification of molecular subtypes, such as CLDN18.2-positive and HER2-negative tumors, has added complexity to understanding the epidemiology of this disease. HER2-negative means that the cancer cells do not overexpress the human epidermal growth factor receptor 2 (HER2) protein. HER2 is a receptor found on the surface of some cancer cells that can promote tumor growth. HER2-negative status rules out the use of HER2-targeted therapies, such as trastuzumab, which are effective in HER2-positive cancers [3,4].

Earlier, such cancers were treated with only platinum-based chemotherapy (e.g., cisplatin, oxaliplatin), fluoropyrimidines (e.g., 5-fluorouracil, capecitabine) or taxanes (e.g., paclitaxel, docetaxel) which were non-targeted and focused on general cytotoxic effects, aiming to slow down cancer’s progression but often led to significant side effects (myelosuppression, mucositis, neuropathy, diarrhea, etc.) without being specific to molecular subtypes like CLDN18.2-positive cancers [5].

Recently, zolbetuximab combined with chemotherapy, such as mFOLFOX6, which is a combination of the drugs leucovorin calcium, fluorouracil, and oxaliplatin or CAPOX, which is a combination of capecitabine and oxaliplatin demonstrated a survival benefit in patients with CLDN18.2-positive and HER-2-negative G/GEJ cancers [6,7]. These clinically significant findings have generated considerable interest in CLDN18.2-targeting therapies. In this review, we summarize the clinical implications of CLDN18.2-positive G/GEJ cancer and CLDN18.2-targeting therapy with zolbetuximab.

Search strategy

To review the drug zolbetuximab for the treatment of unresectable gastric or gastroesophageal junction adenocarcinoma, a search was made in PubMed, EMBASE, Cochrane, Scopus, ResearchGate, UpToDate and Current Oncology using keywords “CLDN18.2”, “Zolbetuximab”, “Chimeric monoclonal antibody”, “Gastric adenocarcinoma”, “Gastroesophageal Adenocarcinoma”, etc. We also reviewed the references from several surveyed articles and compiled the final bibliography accordingly.

Pharmacology of zolbetuximab

Zolbetuximab is a chimeric immunoglobulin G1 monoclonal antibody produced from murine antibodies that binds to isoform 2 of Claudin protein 18 [8]. It is chimerized to resemble the human immunoglobulin G1 constant region for clinical application, and it works by triggering the immune system to attack the cancer cells expressing this protein [8]. It exhibits high sensitivity and specificity for the CLDN18.2 protein expressed on the surface of malignant cells, particularly at the first extracellular loop of CLDN18.2 [9]. Zolbetuximab’s mechanism of targeting Claudin-18.2 represents the first gastric cancer-specific 'targeted' treatment. It attaches to CLDN18.2 on the surface of tumour cells and stimulates both cellular and soluble immune effectors, which in turn trigger antibody-dependent cytotoxicity (ADCC) and complement-dependent cytotoxicity (CDC).

**Figure 1:**
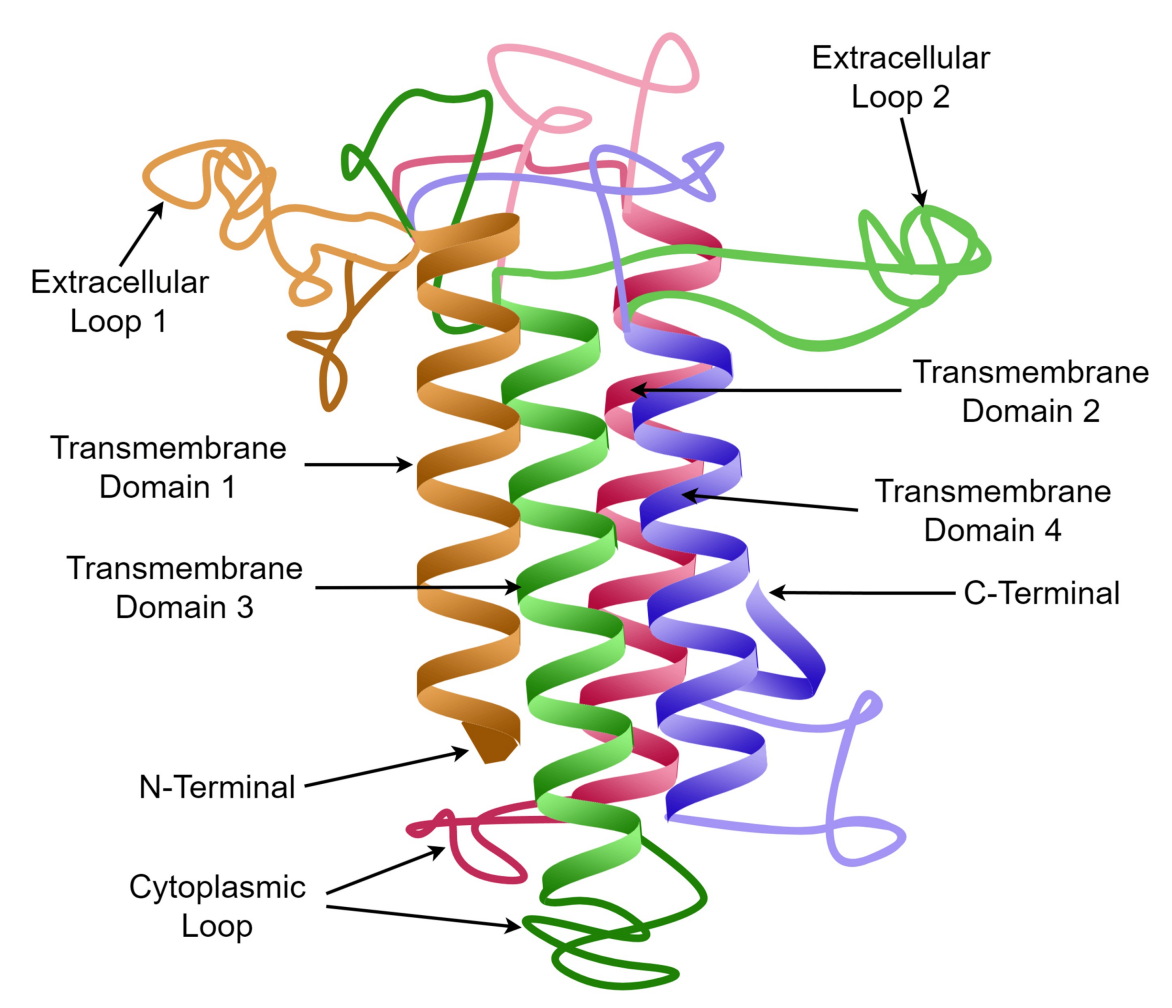
The model structure of CLDN protein. The image is the original work of the authors.

A model structure of the CLDN protein is shown in Figure 1. The structure of CLDN proteins consists of four transmembrane domains, including an N-terminus and C-terminus in the cytoplasm and two extracellular loops. Potential therapeutic antibodies interact with CLDN proteins in the extracellular loops. The C-terminus is the site of phosphorylation and interaction with signaling molecules. These signaling molecules then promote the immune effector's cells to cause apoptosis of CLDN18.2-positive cells [9,10].

Claudin 18 protein, particularly its isoform 2, is expressed only in gastric mucosa in healthy tissues. Its expression is retained during transformation to malignant gastric cancer cells and ectopically expressed in metastatic lesions. It is normally present on the apical side of tight junctions and remains unexposed to the cell surface. It is responsible for maintaining epithelial barrier integrity and cell adhesion and communication. Malignant transformation of gastric cells leads to exposure of CLDN18 protein on the cell surface due to damage in cell polarity. Alterations in CLDN proteins, such as a reduced expression or fusion with other GTP-activating proteins, contribute to tumour development and proliferation [8,9]. Abnormal Claudin expression can disrupt tight junction functionality, affect signalling pathways, and serve as a tumour-promoting factor in certain epithelial cancers.

**Figure 2:**
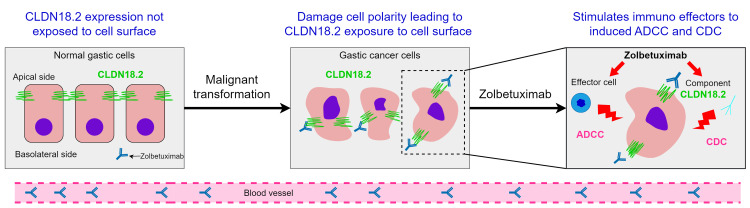
Working mechanism of zolbetuximab. This image has been taken from an open access article distributed under the terms of the Creative Commons Attribution License, which permits unrestricted use, distribution, and reproduction in any medium, provided the original author and source are credited [7].

Anti-Claudin antibodies (such as zolbetuximab and claudiximab) attach to CLDN18.2 on the surface of tumour cells, stimulating cellular and soluble immune effectors to activate ADCC and CDC [7]. Both ADCC and CDC contribute to zolbetuximab's antitumor activity. In ADCC, zolbetuximab attaches to CLDN18.2 on cancerous cells, recruiting natural killer cells through Fc gamma receptors (FcγR) located on its Fc region. This interaction initiates the release of cytotoxic granules, such as perforin and granzymes, which lead to apoptosis in the targeted cells. In CDC, zolbetuximab's binding to CLDN18.2 activates the classical complement pathway by allowing the C1 complex to bind to the antibody’s Fc region. This interaction triggers a proteolytic cascade that ultimately results in the formation of the membrane attack complex (MAC), causing disruption and lysis of the cancer cell membrane [7]. When used in conjunction with fluoropyrimidine and platinum-based chemotherapy agents, zolbetuximab can also promote T-cell infiltration and stimulate the release of proinflammatory cytokines. This elucidates the mechanism by which zolbetuximab acts alongside fluoropyrimidine and platinum-containing chemotherapy as a first-line treatment for CLDN18.2-positive G/GEJ cancer (Figure 2) [7,10,11].

Pharmacodynamics of zolbetuximab

Dosage

Zolbetuximab is typically administered intravenously. Phase III clinical trials, such as the SPOTLIGHT study, reveal that the initial dose is usually higher to achieve optimal target saturation, followed by maintenance doses. Typically, the Loading dose is 800 mg/m² on Day 1 of the treatment cycle, and subsequent doses are 600 mg/m² on Days 8 and 15 of Cycle 1, and then every two weeks (Days 1 and 15) for subsequent cycles [11]. However, the exposure-response relationships for safety and efficacy at recommended doses of zolbetuximab are yet to be fully characterized.

Pharmacokinetics of zolbetuximab

Mode of Administration

Zolbetuximab is administered via IV infusion over a set duration, particularly for cases of locally advanced unresectable or metastatic gastric and gastroesophageal adenocarcinoma. The first infusion (loading dose) is usually given over several hours, typically two to four hours, under close observation for any infusion-related reactions. Subsequent infusions (maintenance doses) are administered over a shorter time, often one to two hours, depending on patient tolerance. Premedication is commonly administered to prevent infusion-related reactions. This may include antihistamines, corticosteroids, and antipyretics to reduce risks of hypersensitivity reactions and other side effects like chills, fever, or nausea [11,12].

Studies have shown that following a two-hour intravenous infusion, zolbetuximab exhibited dose-proportional pharmacokinetics at doses ranging from 33 mg/m^2^ to 1000 mg/m^2^ (0.04 times to 1.25 times the recommended first dose). This means that the drug concentration in the blood increases proportionally with the dose, making its pharmacokinetic profile predictable [12,13]. When administered at a first dose of 800 mg/m^2^ followed by subsequent doses of 600 mg/m^2^ every three weeks, steady state was achieved by 18 weeks, that is, usually by the third or fourth infusion during a treatment cycle with a geometric mean (coefficient of variation [CV]%) Cmax of 415 (22%) mcg/mL and AUC tau of 3149 (37%) day•mcg/mL. The modest accumulation ratio from the first dose to the steady state ensures sustained plasma levels to maintain efficacy without requiring frequent administration. The drug is metabolized into small peptides and amino acids [14].

Elimination Half-Life

In clinical trials, the pharmacokinetic profile of zolbetuximab revealed that it follows a linear pattern, with a mean elimination half-life of about 17 days consistent across various dosages, as observed in the phase 1 dose-escalation study (NCT00909025), where patients received doses ranging from 33 mg/m² to 1000 mg/m² without dose-limiting toxicities, and the drug was generally well-tolerated [15]. The long half-life, similar to studies in other monoclonal antibodies (ranging from 11 to 14 days) [9], supports dosing intervals every two to three weeks, typically using an 800 mg/m² loading dose followed by 600 mg/m² every three weeks. This slow clearance allows for sustained therapeutic exposure and efficient maintenance of therapeutic levels, minimizing the frequency of administration required to achieve efficacy.

Metabolism

 Zolbetuximab undergoes clearance primarily through catabolic degradation rather than conventional renal or hepatic excretion pathways. Monoclonal antibodies, including zolbetuximab, are largely processed by cells in the reticuloendothelial system (RES), such as macrophages and liver cells, where they undergo internalization followed by lysosomal degradation into peptides and amino acids. This pathway is advantageous as it minimizes dependency on renal or hepatic function, suggesting that zolbetuximab’s pharmacokinetics are less likely to be impacted in patients with compromised kidney or liver function. The gradual catabolic breakdown of zolbetuximab also contributes to its extended half-life and sustained therapeutic presence allowing its less frequent dosing schedule [16].

## Review

Therapeutic efficacy of zolbetuximab

Zolbetuximab has undergone several trials to judge its therapeutic efficacy and safety profile. Some major trials are summarized in Table 1. The different trials and their outcomes are briefly reported in the following section.

**Table 1:** Summary of the trials. PFS: progression-free survival, OS: Overall survival, ADCC:antibody-dependent cellular cytotoxicity, G: gastric and GEJ: gastroesophageal junction adenocarcinomas.

Trial	Aim	Outcome
Phase I (NCT000909025) 15 Japanese patients with gastric cancer [15].	To assess the prevalence of CLDN18.2 in primary tumors and lymph node metastases.	The drug was well-tolerated, with side effects such as nausea and vomiting being manageable.
Phase I (NCT00909025) 35 patients with G/GEJ cancers from Japan [16].	To determine the dose-limiting toxicities.	There were no dose-limiting toxicities observed. The median progression-free survival (PFS) and overall survival (OS) reported were 2.6 months and 4.4 months, respectively.
Phase I (NCT01671774) 28 patients with previously treated advanced G/GEJ [17].	The activity of ADCC and the effects of the immune-modulating agents zoledronic acid and interleukin-2 when combined with zolbetuximab.	The drug effectively mediates ADCC in patients with pretreated advanced G/GEJ cancer.
Phase II (MONO) (NCT01197885) 28 patients with CLDN18.2-positive G/GEJ cancer [18].	To zolbetuximab as monotherapy	Disease control rate (DCR) was observed in a significant proportion of patients, though monotherapy efficacy was moderate compared to combination regimens
Phase II (FAST) (NCT01630083) 262 patients with primary gastric tumors and LN metastases [19, 20].	The effectiveness and safety of zolbetuximab when combined with EOX chemotherapy in patients with advanced G/GEJ cancer.	Both PFS at 7.5 months and OS at 8.3 months showed significant improvement with zolbetuximab plus EOX compared to EOX alone.
Phase II (ILUSTRO) (NCT03505320) 54 patients with CLDN18.2-positive advanced/metastatic G/GEJ adenocarcinoma [21,22].	To evaluate the efficacy and safety of zolbetuximab, either as a standalone treatment or in combination with mFOLFOX6 or pembrolizumab.	The combination of zolbetuximab and mFOLFOX6 showed encouraging efficacy in patients with CLDN18.2-positive G/GEJ cancer who had not been previously treated.
Phase III (SPOTLIGHT) (NCT03504397) 565 patients from 215 centers with G/GEJ cancer [23].	To study the cost-effectiveness and survivability when treated with zolbetuximab and its combinations.	Zolbetuximab significantly extends survival when combined with chemotherapy.
Phase III (GLOW) (NCT03653507) [12].	To assess the efficacy and safety of zolbetuximab in combination with CAPOX for G/GEJ cancer.	The combination of zolbetuximab with CAPOX provided an additional therapeutic option with comparable efficacy to mFOLFOX6

Phase I Trials

Different researchers carried out several Phase I trials to study safety and dosing considerations, and the outcomes of the trials are reported below.

In the trial NCT000909025, each of the 15 patients received a single infusion from one of five dose groups (33, 100, 300, 600, and 1,000 mg/m^2^) of zolbetuximab [15]. This demonstrated a linear pharmacokinetic profile with a half-life of 17 days. No life-threatening adverse events were reported with single doses up to 1,000 mg/m^2^. One patient who received 600 mg/m^2^ maintained stable disease at the four-week follow-up. Due to the limited sample size, no conclusive findings could be established regarding the potential relationship between CLDN18.2 expression levels and the efficacy of zolbetuximab.

Another trial, NCT00909025, conducted in Japan, evaluated patients with CLDN18.2-positive, previously treated locally advanced or metastatic gastric/GOJ cancer, with disease progression on previous lines of therapy [16]. 35 patients received 800 mg/ m^2^ intravenous zolbetuximab on cycle one, day one, followed by 600 mg/m^2^ or 1,000 mg/m^2^ every three weeks. Similar to the previous phase I study, this trial did not identify any dose-limiting toxicities. The best overall response was stable disease (SD) in 64.7% and progressive disease (PD) in 35.3%, yet only two patients had SD lasting more than six months. The median progression-free survival (PFS) and overall survival (OS) durations were 2.6 months and 4.4 months, respectively.

A third Phase I trial (NCT01671774) was engineered to induce ADCC and CDC. The trial evaluated the activity of ADCC, the effects of the immune-modulating agents zoledronic acid (ZA) and interleukin-2 (IL-2) when used in conjunction with zolbetuximab, focusing on pertinent immune cell populations and ADCC lysis activity in patients with advanced or metastatic G/GEJ cancer [17]. A total of 28 patients were assigned to four treatment groups. Treatment with zolbetuximab, zoledronic acid (ZA), and interleukin-2 (IL-2) led to a brief expansion and activation of ADCC-mediating cell populations, specifically γ9δ2 T cells, and natural killer cells, within two days post-administration, with this effect being more significant at the intermediate dose of IL-2. The expansion and activation of regulatory T cells treated with either dose of IL-2 were moderate and transient. Notably, strong ADCC activity was seen with zolbetuximab alone. Several patients receiving zoledronic acid (ZA) with intermediate-dose IL-2 exhibited short-lived ADCC activity, which was not observed with lower-dose IL-2. In the population assessed for clinical efficacy, the most favourable confirmed response was a stable disease, observed in 11 out of 19 patients (58%). Although this combination therapy was generally well tolerated, it did not show additional improvements compared with zolbetuximab alone.

Conclusion: The early Phase I studies established zolbetuximab's safety and determined its maximum tolerated dose. These studies demonstrated that zolbetuximab was well-tolerated, with manageable side effects like nausea and vomiting likely due to the expression of CLDN18.2 in the gastric lining.

Phase II Trials

Phase II trials primarily targeted zolbetuximab’s efficacy and response rates. The outcomes of the different research are given below.

In a multicenter, phase IIa clinical trial called the MONO study (NCT01197885) [18], zolbetuximab was administered as monotherapy to patients with advanced CLDN18.2-positive gastric and gastroesophageal junction adenocarcinomas. The trial evaluated the efficacy of zolbetuximab as a single agent in patients whose tumors exhibited moderate to strong CLDN18.2 expression in 50% or more of cancer cells. Seventy-two patients received zolbetuximab biweekly at two dosage levels: 300 mg/m² for a safety run-in and 600 mg/m² as the target dose. Clinical benefit was observed in 23% of patients, with four experiencing a partial response and six maintaining stable disease.

Another randomized, multicenter phase IIb clinical trial, known as the FAST study (NCT01630083) [19], evaluated the efficacy of zolbetuximab in combination with epirubicin, oxaliplatin, and capecitabine (EOX) (arm 1) compared to EOX alone (arm 2) as a first-line treatment for patients with advanced gastric and gastroesophageal junction adenocarcinoma exhibiting moderate to strong CLDN18.2 expression in 40% or more of tumour cells, involving a total of 263 patients. Both PFS (HR=0.44; 95% CI, 0.29-0.67; p< 0.0005) and OS(HR=0.55; 95% CI, 0.39-0.77; p<0.0005) showed significant improvement with the combination of zolbetuximab and EOX (arm 2) compared to EOX alone (arm 1). This notable benefit in PFS was also observed in patients with moderate to strong CLDN18.2 expression in 70% or more of tumour cells (HR=0.38; 95% CI, 0.23-0.62; p<0.0005). This result aligns with findings from the MONO study [18], where nine out of ten patients who experienced clinical benefit had moderate to strong CLDN18.2 expression in 70% or more of their tumour cells, suggesting a possible correlation between CLDN18.2 expression and the efficacy of zolbetuximab.

Another multicohort phase II trial, the ILUSTRO study (NCT03505320) [20, 21], examined zolbetuximab both as a monotherapy and in combination with modified FOLFOX6 (mFOLFOX6), with or without nivolumab or pembrolizumab. Patients received zolbetuximab as monotherapy in third or later lines (Cohort 1A, n=30), combined with mFOLFOX6 in first-line treatment (Cohort 2, n=21), or alongside pembrolizumab in third or later lines (Cohort 3A, n=3). Results from ILUSTRO Cohort 2, where patients were treated with zolbetuximab plus mFOLFOX6, showed a median PFS of 13.7 months (95% Confidence Interval: 7.4-not estimable), a 12-month PFS rate of 58%, and an objective response rate of 63.2% (95% CI: 38.4-83.7).

Conclusion*:* Zolbetuximab demonstrated antitumor activity as a single agent. Disease control rate (DCR) was observed in a significant proportion of patients, though monotherapy efficacy was moderate compared to combination regimens. The safety profile remained consistent with the first-in-human studies (nausea, vomiting, fatigue), confirming tolerability at the recommended doses [23].

Phase III Trials

A series of Phase III clinical trials had been conducted with the primary objective of survivability when treated with zolbetuximab and combined chemotherapy.

The phase III studies SPOTLIGHT (NCT03504397) were designed as potential registration trials to assess the advantages of adding zolbetuximab to chemotherapy (either mFOLFOX6 or capecitabine and oxaliplatin (CAPOX)) in previously untreated patients with recurrent or metastatic HER-2-negative, CLDN18.2-positive G/GEJ cancer, defined as having moderate to strong CLDN18.2 expression in 75% or more of tumour cells [11]. In the SPOTLIGHT trial, 565 patients from 215 centers were enrolled and randomly assigned (1:1) to either zolbetuximab plus mFOLFOX6 or placebo plus mFOLFOX6. Zolbetuximab 800 mg/m^2^ was administered on cycle 1, day 1, followed by 600 mg/m^2^ on cycle one, day 22, and every three weeks in the following cycles. Conversely, mFOLFOX6 was administered on days 1, 15, and 29 for four 42-day cycles. Treatment with zolbetuximab led to a significant 25% decrease in the risk of disease progression (HR=0.75, 95% CI 0.60-0.94; p=0.0066) and extended the median PFS to 10.61 months (95% CI 8.90-12.48), compared to 8.67 months in the placebo group (95% CI 8.21-10.28). Moreover, the median OS was significantly longer in the zolbetuximab group compared to the placebo group, with median durations of 18.23 months versus 15.54 months (HR=0.75, 95% CI 0.60-0.94; p=0.0053). The objective response rate (ORR) was comparable between the two treatment groups, at 61% and 62% for patients with measurable disease. Progression-free survival was 10.6 months in the zolbetuximab+mFOLFOX6 group, compared to 8.7 months in the placebo+mFOLFOX6 group. Additionally, OS was enhanced, with OS at 14.6 months for the zolbetuximab group compared to 12.4 months for the placebo group. Notably, subgroup analysis showed that patients with the diffuse intestinal Lauren subtype had significantly longer PFS and OS when treated with zolbetuximab. Meanwhile, no additional survival benefits were observed amid patients with mixed and other Lauren subtypes. Additionally, patients with GEJ cancer experienced lower benefits of zolbetuximab compared with gastric cancer patients. Yet, a cautious interpretation of these findings is warranted due to the small GEJ cancer subgroup, and future trials should recruit more patients with GEJ cancer to withdraw conclusions precisely.

The cost of the drug was determined based on national bid prices, while other expenses and utility values were sourced from the published literature. The outcomes measured included total costs, quality-adjusted life years (QALYs), and incremental cost-effectiveness ratios (ICERs). The robustness of the model was assessed through one-way sensitivity and probabilistic sensitivity analyses. The treatment group achieved 1.64 QALYs at a cost of $87,746.35, whereas the placebo group achieved 1.23 QALYs at a cost of $11,947.81. The ICER for the treatment compared to the placebo group was $185,353.28 per QALY gained. These results indicate that the combined treatment is unlikely to be cost-effective as a first-line treatment for CLDN18.2-positive, HER2-negative advanced G/GEJ adenocarcinoma [22].

Another phase III trial, known as GLOW (NCT03653507) [23], was conducted to evaluate the efficacy and safety of combining zolbetuximab with CAPOX chemotherapy compared to CAPOX with placebo. Patients received an 800 mg/m² loading dose of zolbetuximab on the first day of cycle one, followed by 600 mg/m² every three weeks. The CAPOX regimen included 130 mg/m² of oxaliplatin administered intravenously on day one and oral capecitabine at a dose of 1,000 mg/m² taken twice daily on days 1-14 of each cycle for a total of eight 21-day cycles. The combination of zolbetuximab and CAPOX demonstrated a statistically significant improvement in PFS, successfully achieving the primary endpoint of the trial. Patients receiving zolbetuximab experienced a 31.3% reduction in the risk of disease progression or death compared to the placebo group (HR= 0.687, 95% CI 0.544-0.866; p=0.0007). Furthermore, the zolbetuximab group showed significant enhancements in median PFS (8.21 months versus 6.80 months; HR=0.687; p=0.0007) and OS (14.39 months versus 12.16 months; HR=0.771, p=0.0118) compared to the placebo group. Among patients with measurable disease, the ORR was 53.8% for the zolbetuximab group compared to 48.8% for the placebo arm.

Conclusion*:* The two large Phase III studies showed a significant improvement in PFS and OS when treated with two widely accepted standard-of-care chemotherapy regimens for G/GEJ adenocarcinomas. With OS benefit, zolbetuximab in combination with CAPOX is comparable to the trial with mFOLFOX6. Additionally, the GLOW trial exhibited an earlier separation of the survival curves than the SPOTLIGHT trial, indicating a potentially more favorable outcome for the CAPOX plus zolbetuximab treatment regimen.

Till the writing of this review, several exploratory trials under Phase IV are going on investigating zolbetuximab in other cancer types and in combination with different treatment regimens, for example, combinations with immunotherapy agents, such as PD-1 inhibitors. Further studies are likely to focus on biomarker-driven stratification, refining the selection of patients based on CLDN18.2 expression levels, and exploring combination therapies that might further improve outcomes [24].

Black box warning

The black box warnings for zolbetuximab, particularly in trials involving patients with HER2-negative and CLDN18.2-positive G/GEJ adenocarcinoma, focus on potential serious side effects that have been observed in clinical studies [24]. These warnings are highlighted to alert both patients and healthcare providers to risks associated with this targeted monoclonal antibody therapy. As of the latest data from clinical trials, here are some of the key warnings and serious adverse effects associated with zolbetuximab [24].

Severe Gastrointestinal Toxicity

Zolbetuximab targets CLDN18.2, and it is also found in normal gastric cells. This dual targeting may lead to gastrointestinal adverse effects, such as nausea, vomiting, and abdominal pain, which can sometimes be severe.

Infusion-Related Reactions

Like many monoclonal antibodies, Zolbetuximab has been associated with infusion-related reactions (IRRs), which may include fever, chills, rash, low blood pressure, and difficulty breathing. Pre-medications and close monitoring during infusions are typically recommended to mitigate these reactions.

Severe Hypersensitivity Reactions

Hypersensitivity or allergic reactions have been observed and may require immediate medical intervention. This includes symptoms like swelling, difficulty breathing, and skin reactions, which can be life-threatening if not promptly managed.

Hepatotoxicity

There is potential for liver-related adverse effects, which can include elevated liver enzymes or even severe hepatotoxicity in rare cases. Monitoring of liver function tests is often recommended during therapy.

Cytokine Release Syndrome

Although less common, Cytokine release syndrome (CRS) has been observed in some patients, which can result in fever, hypotension, and multi-organ dysfunction. CRS is a serious complication that often requires immediate treatment, including immunosuppressive therapy.

Meta-analysis for different standards of care

In a study, a meta-analysis was conducted to evaluate the efficacy of the anti-Claudin-18.2-targeted therapy, zolbetuximab, in comparison to other anti-programmed cell death-1/programmed cell death-ligand 1 (PD-1/PD-L1) inhibitors for treating gastric/gastroesophageal junction (G/GEJ) carcinoma [22]. This study included eight trials with 6,455 patients diagnosed with gastric and gastroesophageal junction adenocarcinomas. The therapeutic agents compared were pembrolizumab, sintilimab, sugemalimab, tislelizumab, and nivolumab. Table 2 provides a summary of the meta-analysis results [22].

**Table 2:** Summary of meta-analysis results. PFS: progression-free survival, OS: Overall survival, ORR: overall response rate.

Analysis	Therapy	HR	95% CI	Result
OS	pembrolizumab	1.00	0.94-1.07	No statistically significant differences were observed between zolbetuximab and other inhibitors
	sintilimab	0.99	0.89-1.09	
	sugemalimab	0.98	0.87-1.10	
	tislelizumab	0.97	0.87-1.09	
	zolbetuximab	0.98	0.91-1.07	
	nivolumab	1.00	-	
PFS	pembrolizumab	1.00	0.93-1.06	No statistically significant differences were observed between zolbetuximab and other inhibitors
	sintilimab	0.91	0.83-1.00	
	sugemalimab	0.92	0.84-1.02	
	tislelizumab	0.93	0.84-1.03	
	zolbetuximab	0.96	0.88-1.05	
	nivolumab	1.00	-	
ORR				All regimens presented similar effects on ORR

It may be noted that anti-Claudin-18.2-targeted therapies presented similar OS (HR=0.99, 95% CI: 0.95-1.04) and PFS (HR=1.01, 95% CI: 0.91-1.12) compared to immunotherapy, although their toxicity profiles were distinct [24].

Synopsis

The meta-analysis [22] revealed no significant differences in progression-free survival (PFS), overall survival (OS), or overall response rate (ORR) among various checkpoint inhibitors or between immunotherapy and anti-Claudin-18.2-targeted therapies for first-line treatment of HER2-negative, unresectable, or metastatic gastric cancers.

## Conclusions

Zolbetuximab is a first-in-class investigational monoclonal antibody targeting CLDN18.2 to the best of our knowledge. The United States FDA has approved this drug following the biologics license application submission for zolbetuximab as a first-line treatment for locally advanced unresectable or metastatic HER2-negative G/GEJ adenocarcinoma with Claudin 18.2 positive tumours. The biologics license application submission is supported by the phase III SPOTLIGHT and GLOW studies. Zolbetuximab is also the first CLDN18.2-targeted therapy, to the best of our knowledge, to receive approval for this patient population in the U.S. and other countries. This innovative targeted therapy represents a significant advancement for patients with challenging G/GEJ cancers. Individuals living with advanced G/GEJ cancer often encounter significant unmet needs, and the FDA approval of zolbetuximab moves us closer to providing this crucial treatment option to eligible patients battling this serious disease.
